# iTRAQ-based proteomics analysis of *Bacillus pumilus* responses to acid stress and quorum sensing in a vitamin C fermentation system

**DOI:** 10.3389/fmicb.2023.1131000

**Published:** 2023-03-21

**Authors:** Qian Zhang, Shuxia Lyu

**Affiliations:** College of Bioscience and Biotechnology, Shenyang Agricultural University, Shenyang, Liaoning, China

**Keywords:** microbial consortium, quorum sensing, proteomics, communication, vitamin C

## Abstract

Microbial consortia play a key role in human health, bioenergy, and food manufacturing due to their strong stability, robustness and versatility. One of the microbial consortia consisting of *Ketogulonicigenium vulgare* and *Bacillus megaterium* for the production of the vitamin C precursor, 2-keto-L-gulonic acid (2-KLG), has been widely used for large-scale industrial production. To further investigate the cell–cell communication in microbial consortia, a microbial consortium consisting of *Ketogulonicigenium vulgare* and *Bacillus pumilus* was constructed and the differences in protein expression at different fermentation time points (18 h and 40 h) were analyzed by iTRAQ-based proteomics. The results indicated that *B. pumilus* was subjected to acid shocks in the coculture fermentation system and responded to it. In addition, the quorum sensing system existed in the coculture fermentation system, and *B. pumilus* could secrete quorum-quenching lactonase (YtnP) to inhibit the signaling pathway of *K. vulgare*. This study offers valuable guidance for further studies of synthetic microbial consortia.

## 1. Introduction

In natural environments, microorganisms normally exist as communities of multiple species, capable of performing a variety and complexity of tasks and different members of a consortium assuming different responsibilities, increasing a higher overall productivity and allowing a more complex behavior than that of a monoculture (Brune and Bayer, 2012; Wang et al., 2016). With the development of synthetic communities, microbial consortia have become increasingly important in industrial applications (Jia et al., 2016). Therefore, there is an increasing focus on the development of synthetic consortia in industry. In general, several types of microbial interactions, i.e., food competition, mutualism, amensalism, synergism, predation, and parasitism could be involved in a mixed culture fermentation process (Sabra et al., 2010). However, microbial consortia in industry are complex, as the exchange of biomolecules (e.g., proteins, nucleic acids and metabolites) and information signals with the surroundings have to be taken into consideration, and the relationships among the strains are often too diverse to analyze (Wang et al., 2016; Ma et al., 2019; Roell et al., 2019).

Here, an example is described of using microbial consortia in industry for the production of vitamin C (L-ascorbic acid) to reduce costs and increase product quality. Microbial consortia of *Ketogulonicigenium vulgare* and *Bacillus* spp. have been widely used in a two-step vitamin C fermentation process and have achieved great economic benefits (Zhang and Lyu, 2022). *K. vulgare* is responsible for producing 2-keto-L-gulonic acid (2-KLG, the precursor of vitamin C) from L-sorbose *via* L-sorbosone but with an extremely low yield. *Bacillus* strains (e.g., *Bacillus megaterium*, *Bacillus endophyticus*, *Bacillus subtilis*, *Bacillus pumilus*), as helper strains, are cocultured to significantly stimulate the growth of *K. vulgare* and 2-KLG yields (Yakushi et al., 2020; Zeng et al., 2020; Fang et al., 2021; Yang et al., 2021; Zhang et al., 2022). The relationship between *K. vulgare* and the helper strain has been one of the hot topics focused on in the field of vitamin C fermentation.

In recent years, the interaction and communication between *K. vulgare* and the helper strain have been well explained with the development and application of omics technology (e.g., proteomics, metabolomics, and genomics) (Ma et al., 2011; Zhou et al., 2011; Jia et al., 2015; Ma et al., 2019; Zhang and Lyu, 2022). Most of the relevant studies have illustrated that the interaction between the two bacteria is a synergistic combination of mutualism and antagonism (Zhou et al., 2011). The helper strain provides key elements necessary to promote the growth and 2-KLG production of *K. vulgare*, whereas *K. vulgare* may accelerate the sporulation of the helper strain (Ma et al., 2011). In addition, Zhang et al. (2015) used Monod-type equations to describe the interactions between the two bacteria, which showed that *K. vulgare* was a predator and that the helper strain was prey. Moreover, it has been reported that L-sorbose, the substrate for 2-KLG production, not only promotes the growth of *K. vulgare* but also inhibits the growth of the helper strain, and the helper strain that is L-sorbose-tolerant enhances 2-KLG productivity (Mandlaa et al., 2018). Although it seems that the growth of the helper strain is inhibited in the coculture fermentation system, the cell–cell communication and the specific biomolecules that inhibit the growth of the helper strain are still not clear.

In this paper, our aim was to investigate the cell–cell communication and the reasons for inhibiting the growth of the helper strain in the coculture fermentation system. We therefore hypothesized that *K. vulgare* produced signals in the environment and that the oxidization products, including 2-KLG, could inhibit the growth of the helper strain. To test these hypotheses, iTRAQ-based proteomics analysis of an artificial consortium of *K. vulgare* and *B. pumilus* was performed, and the results used to derive novel information for further understanding of the synthetic consortium in Vc microbial fermentation and hopefully shed light on other complex microbial consortia.

## 2. Materials and methods

### 2.1. Strains and media

*Ketogulonicigenium vulgare* 25B-1 (*K. vulgare* 25B-1) and *Bacillus pumilus* SY-A9 (*B. pumilus* SY-A9) were kindly provided by Northeast Pharmaceutical Group Co. Ltd. (Shenyang, China). These strains were stored at −80°C in glycerol.

Three media were used. Isolation medium: 20 g/L L-sorbose (autoclaved separately), 10 g/L peptone, 1 g/L urea, 5 g/L corn-steep liquor, 3 g/L yeast extract, 3 g/L beef extract, 1 g/L KH_2_PO_4_, 0.4 g/L MgSO_4_, 1 g/L CaCO_3_, 20 g/L agar powder, pH 6.7. The seed medium was the same as the isolation medium without the addition of agar powder. Fermentation medium: 80 g/L L-sorbose (autoclaved separately), 12 g/L urea, 10 g/L corn-steep liquor, 0.2 g/L MgSO_4_, 1 g/L KH_2_PO_4_, 5 g/L CaCO_3_, pH 6.7. These media were autoclaved at 121°C for 15 min before use.

### 2.2. Seed preparation and fermentation in flasks

First, approximately 1,000 colonies of *K. vulgare* were collected from the isolation medium plate (*K. vulgare* had been incubated for 48 h at 28°C) and suspended in 2 mL of sterile water. Second, one colony of *B. pumilus* with a diameter of 1 mm from the isolation medium plate (*B. pumilus* had been incubated for 24 h at 28°C) was mixed with 2 mL of sterile water. Then, 1 mL of *B. pumilus* solution and 2 mL of *K. vulgare* solution was mixed and the mixture was inoculated into a 20-mL seed culture medium in a 250-mL flask. After shaking (170 rpm) for 16 h at 28°C, the seeds were obtained, and *K. vulgare* colony number reached 1 × 10^9^ CFU/mL and *B. pumilus* colony number reached 1 × 10^6^ CFU/mL. Subsequently, 2 mL of seed was inoculated into a 20-mL fermentation medium in a 250-mL flask and the flask was cultivated on a shaker with 170 rpm at 28°C.

### 2.3. Sample preparation and iTRAQ assays

The fermentation broths at 18 h and 40 h were centrifuged at 13,000 × *g* for 20 min to obtain 0.5 g of wet bacteria for protein extraction. Each sample was mixed with 300 μl of breaking buffer (consisting of 50 mM Na_3_PO_4_, 1 mM phenylmethanesulfonyl fluoride (PMSF), 1 mM ethylenediaminetetraacetic acid (EDTA) and 5% glycerol) and complete protease inhibitor cocktail (4,693,116,001; Roche) on ice, followed by centrifugation at 4°C and 15,000 × *g* for 15 min. The supernatant containing intracellular proteins was further quantified for protein concentration using the Bradford assay. 200 μg protein was mixed with 5 mL of 1 M dithiothreitol (DTT) at 37°C for 1 h and alkylated with 20 μL of 1 M indole acetic acid (IAA) at room temperature for 1 h in the dark. Then, trypsin digestion (protein:trypsin ratio of 20:1) was performed for 24 h at 37°C. Using an iTRAQ Reagent-8Plex Multiplex Kit (AB SCIEX), peptides from the four samples were labeled with the iTRAQ tags as follows: *K. vulgare* + *B. pumilus*18 (tags 113 and 114) and *K. vulgare* + *B. pumilus*40 (tags 115 and 116), respectively.

The peptides were further fractionated using a C18 column (4.6 mm × 250 mm, 300 Å) with a high-performance liquid chromatography (HPLC) system (Agilent Technologies, United States) at a flow rate of 0.2 mL/min. The peptides were suspended in mobile phase A (100% H_2_O, pH 10.0, consisting of 20 mM NH_4_Ac) and eluted at a flow rate of 0.2 mL/min with the following gradient: 5 to 35% mobile phase B (90% acetonitrile (ACN), pH 10.0, consisting of 20 mM NH_4_Ac) for 30 min, 35 to 90% mobile phase B for 5 min, 90% mobile phase B for 10 min. The elution peak was monitored at 214 nm and one component per minute was collected and then lyophilized.

### 2.4. Nano LC–MS/MS analysis

The lyophilized peptides were resuspended in 5 μl of 0.5% formic acid (FA), transferred to a C18 trap column (100 μm × 2 cm, 3 μm), and then separated using an Easy nLC 1,000 system with a C18 column (75 μm × 15 cm, 3 μm) at a flow rate of 200 nl/min. The mobile phases were solvent A (0.1% FA) and solvent B (0.1% FA in ACN). The elution gradient was as follows: 0–1 min, 1–5% solvent B; 1–6 min, 5–10% solvent B; 6–85 min, 10–30% solvent B; 85–87 min, 30–40% solvent B; 87–90 min, 40–90% solvent B; 90–100 min, 90% solvent B; 100–101 min, 90–1% solvent B; 101–120 min, 1% solvent B. The end of the nanoliter liquid-phase separation was connected directly to the Q-Exactive mass spectrometer (Thermo Fisher Scientific, United States) with a set of the following parameters: polarity, positive ion mode; MS scan range, 300–1,800 m/z; MS/MS scan resolution, 17,500; normalized collision energy, 30.

### 2.5. Proteomic data analysis

The acquired raw mass spectrometry data were processed and identified using the Sequest server and Proteome Discoverer (PD) (Thermo Fisher Scientific, United States) software. The spectra extracted using the PD software were searched against the Uniprot-Ketogulonicigenium+vulgare&Uniprot-Organism%3Abacillus+ subtilis database using the Sequest server with the following identification parameters: max missed cleavages, 2; fixed modifications, carbamidomethyl (C), iTRAQ8plex (N-term), iTRAQ8plex (K), iTRAQ8plex (Y); variable modifications, oxidation (M), deamidated (N, Q); peptide mass tolerance, 10 ppm; fragment mass tolerance, 0.02 Da; enzyme, trypsin. Proteins were quantified based on the PD software. In this paper, proteins with two unique peptides, *p*-values <0.05, fold changes ≥1.2 or ≤ 0.83, were identified as differentially expressed proteins (DEPs) between the different *B. pumilus* strains, whereas *p*-values <0.05, fold changes ≥1.1 or ≤ 0.91 were identified as DEPs between the different *K. vulgare* strains. In other words, in *B. pumilus*, the fold changes of the upregulated DEPs were more than 1.2, while those of the downregulated DEPs were less than 0.83. In *K. vulgare*, the fold changes of the upregulated DEPs were more than 1.1, while those of the downregulated DEPs were less than 0.91. For the convenience of expression, *B. pumilus*18 (B18) vs. *B. pumilus*40 (B40) and *K. vulgare*18 (K18) vs. *K. vulgare*40 (K40) were used to compare the proteome of the former with that of the latter to screen DEPs.

### 2.6. Bioinformatics analysis

Hierarchical cluster analysis of DEPs was performed using R package. Clusters of orthologous genes (COG) annotations of DEPs were carried out using NCBI Blast.^1^ The biological pathway levels of DEPs were determined using the KEGG database.^2^ The functional interaction between the DEPs was performed using String^3^ and Cytoscape (3.8.2).

### 2.7. Quantitative PCR

The sample was collected from the coculture fermentation system at 18 h and 40 h of incubation. Total RNA from each sample was extracted with SteadyPure Universal RNA Extraction Kit (AG21017, Accurate Biotechnology, Hunan, Co., Ltd., China). The synthesis of the first-strand cDNA was performed by an Evo M-MLV RT Permix for quantitative PCR (qPCR) (AG11706, Accurate Biotechnology, Hunan, Co., Ltd., China) after measuring the RNA quality and quantity by a BioDrop Spectrophotometer (Biochrom, England). Specific primers of selected DEPs were designed by NCBI Primer-BLAST (Supplementary Table S1), and 16S rRNA was used as the reference gene. The related gene expression level was determined on a CFX96 real-time PCR system (Bio-Rad, United States) with a total 10 μl volume of reaction mixture containing 0.8 μl template DNA, 0.4 μl forward primer (10 μM), 0.4 μl reverse primers (10 μM), 5 μl SYBR Green Premix Pro Taq HS qPCR Kit (AG11701, Accurate Biotechnology, Hunan, Co., Ltd., China), and 3.4 μl sterile purified water. Relative gene expression was calculated using the 2^−ΔΔCT^ method (Livak and Schmittgen, 2001), followed by statistical analysis with GraphPad Prism 9 (GraphPad Software).

## 3. Results

### 3.1. Overview of proteomic data of the two strains

**Figure 1:**
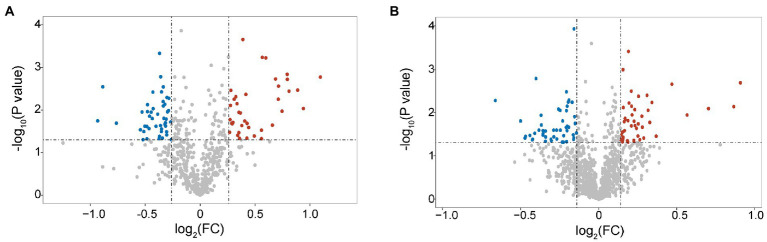
Volcano plot of differentially expressed proteins (DEPs) in *B. pumilus* (B18 vs. B40) **(A)** and *K. vulgare* (K18 vs. K40) **(B)**. Each point represents a protein, and the red and blue areas represent upregulated and downregulated proteins (in *B. pumilus*, FC ≥ 1.2 or FC ≤ 0.83 and *p* < 0.05; in *K. vulgare* FC ≥ 1.1 or FC ≤ 0.91 and *p* < 0.05), respectively.

In total, 1,202 peptides and 584 proteins of *B. pumilus* SY-A9 and 5,475 peptides and 1,134 proteins of *K. vulgare* 25B-1 were obtained *via* iTRAQ labeling and 2D LC-MS/MS analysis in this study. Compared with the control group (samples at 40 h fermentation broth), 82 DEPs (38 upregulated and 44 downregulated) of *B. pumilus* SY-A9 and 96 DEPs (47 upregulated and 49 downregulated) of *K. vulgare* 25B-1 were identified in the 18 h fermentation broth group (Figure 1).

### 3.2. Functional classification of DEPs predicted by COG annotations

**Figure 2:**
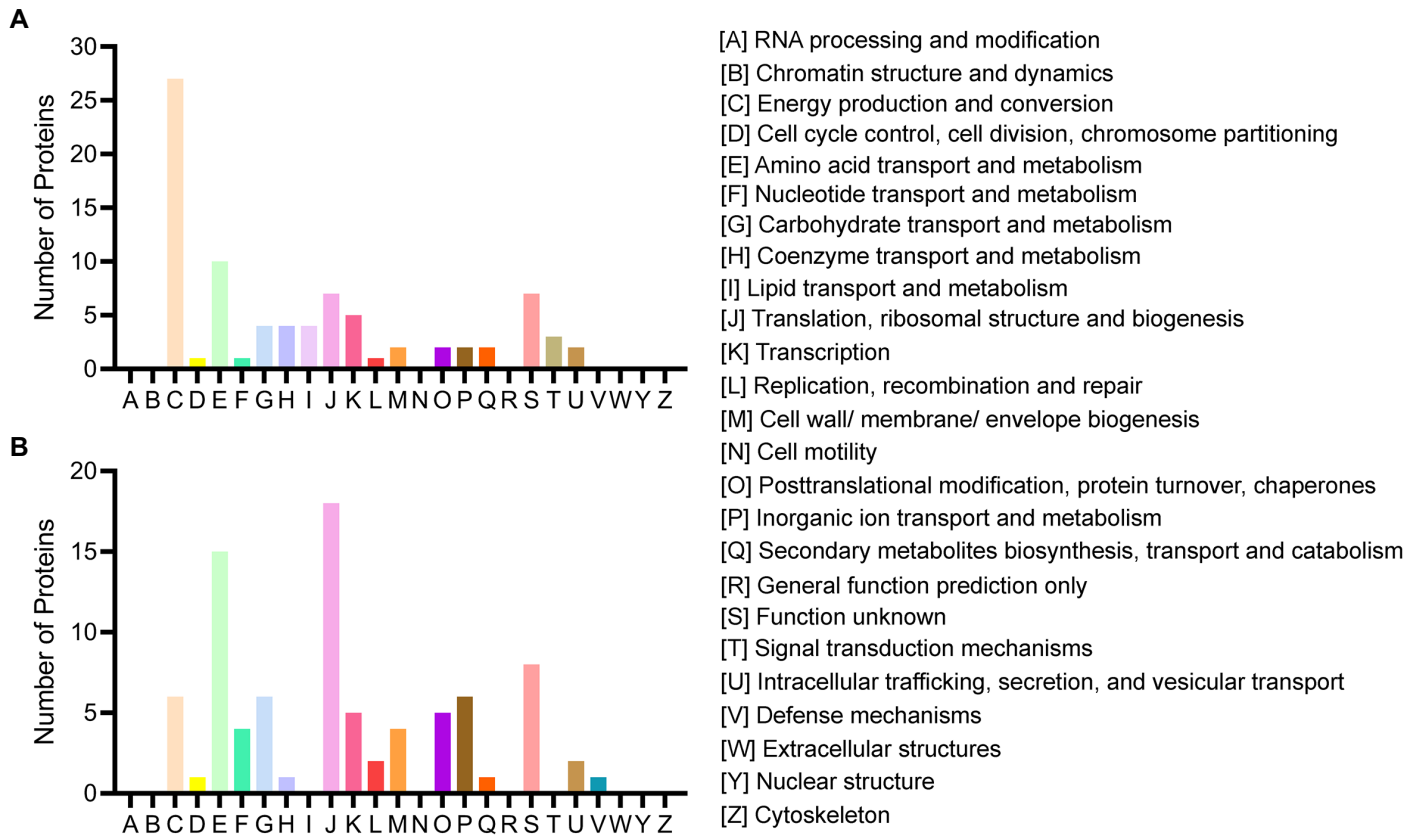
COG functional classification of the DEPs in *B. pumilus* (B18 vs. B40) **(A)** and *K. vulgare* (K18 vs. K40) **(B)**.

All acquired DEPs of *B. pumilus* SY-A9 and *K. vulgare* 25B-1 was subjected to COG functional annotation, and 25 categories were assigned with A-Z representation (Figure 2).

Among all the DEPs of *B. pumilus* SY-A9, five were annotated with multiple functions, and three were not annotated. The five main functional categories were as follows: [C] energy production and conversion (27, 32.14%), [E] amino acid transport and metabolism (10, 11.90%), [J] translation, ribosomal structure and biogenesis (7, 8.33%), [S] function unknown (7, 8.33%), and [K] transcription (5, 5.95%). The detailed functional annotation of the up- and downregulated DEPs of *B. pumilus* SY-A9 was shown in Supplementary Table S2. The obtained DEPs were classified into four main categories, including “information storage and processing,” “cellular processes and signaling,” “metabolism,” and “poorly characterized.” There were 3, 5, 29, and 1 of the upregulated DEPs involved in “information storage and processing,” “cellular processes and signaling,” “metabolism,” and “poorly characterized,” respectively, and 1 had no annotation function. There were 10, 5, 25, and 6 of the downregulated DEPs involved in “information storage and processing,” “cellular processes and signaling,” “metabolism,” and “poorly characterized,” respectively, and 2 had no annotation function.

Among all the DEPs of *K. vulgare* 25B-1, three were annotated with multiple functions, and 14 were not annotated. The three main functional categories were as follows: [J] translation, ribosomal structure and biogenesis (18, 21.18%), [E] amino acid transport and metabolism (15, 17.65%), and [S] function unknown (8, 9.41%). The detailed functional annotation of the up- and downregulated DEPs of *K. vulgare* 25B-1 was shown in Supplementary Table S3. There were 17, 3, 17, and 3 of the upregulated DEPs involved in “information storage and processing,” “cellular processes and signaling,” “metabolism,” and “poorly characterized,” respectively, and 9 had no annotation function. There were 8, 10, 22, and 5 of the downregulated DEPs involved in “information storage and processing,” “cellular processes and signaling,” “metabolism,” and “poorly characterized,” respectively, and 5 had no annotation function.

### 3.3. Pathway enrichment analysis of DEPs by KEGG

**Figure 3:**
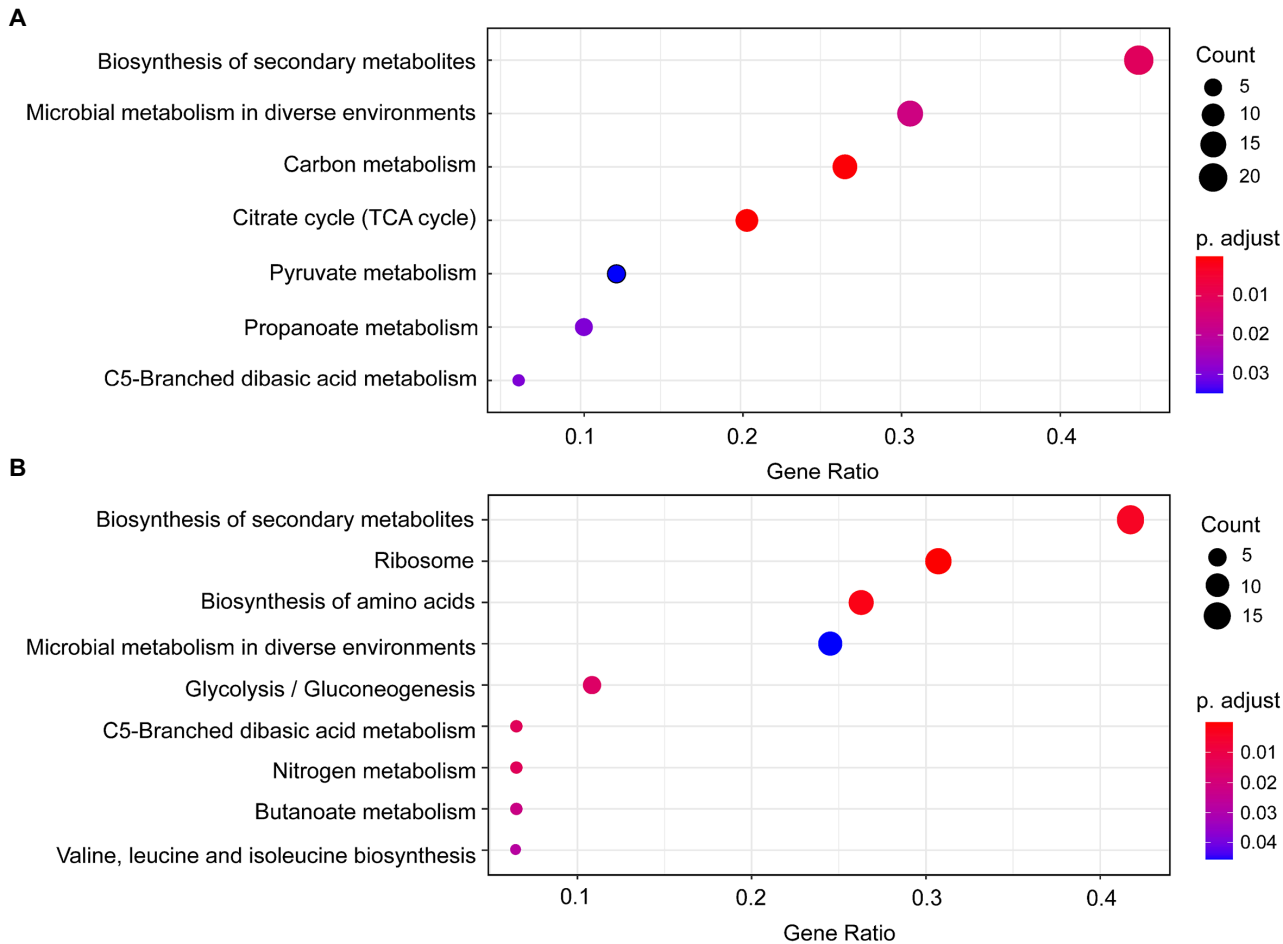
KEGG pathway enrichment of the DEPs in *B. pumilus* (B18 vs. B40) **(A)** and *K. vulgare* (K18 vs. K40) **(B)**. A redder color represents a greater *p-*value, and a larger circle represents a greater number of proteins.

Seven significantly enriched KEGG pathways (*p* < 0.05) were obtained based on the DEPs of *B. pumilus* (B18 vs. B40) (Figure 3A), including biosynthesis of secondary metabolites, microbial metabolism in diverse environments, carbon metabolism, citrate cycle (TCA cycle), pyruvate metabolism, propanoate metabolism, and C5-branched dibasic acid metabolism. Nine significantly enriched KEGG pathways (*p* < 0.05) were acquired based on the DEPs of *K. vulgare* (K18 vs. K40) (Figure 3B), including biosynthesis of secondary metabolites, ribosome, biosynthesis of amino acids, microbial metabolism in diverse environments, glycolysis/gluconeogenesis, C5-branched dibasic acid metabolism, nitrogen metabolism, butanoate metabolism, and valine, leucine and isoleucine biosynthesis.

### 3.4. Interaction network of DEPs

**Figure 4:**
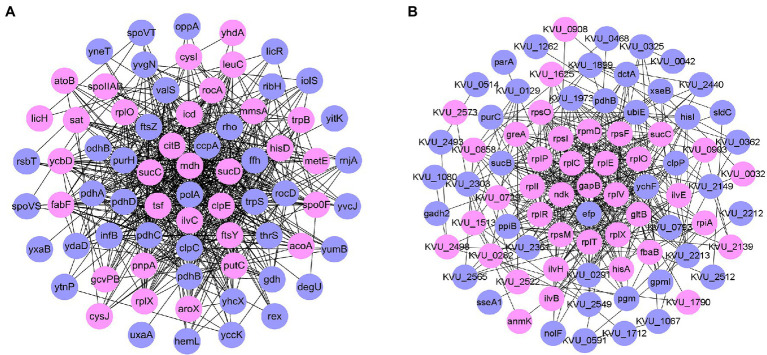
Network analysis of proteins based on differentially expressed proteins (DEPs) in *B. pumilus* (B18 vs. B40) **(A)** and *K. vulgare* (K18 vs. K40) **(B)**. Network analysis revealing the protein–protein interactions among upregulated proteins (pink circle) and downregulated proteins (blue circle).

Protein–protein interaction (PPI) network analysis (Figure 4A) showed that the number of downregulated proteins in *B. pumilus* SY-A9 was higher than that of the upregulated proteins, and the core proteins were mostly related to energy production and conversion.

PPI network analysis (Figure 4B) showed that, the number of downregulated proteins in *K. vulgare* 25B-1 was higher than that of the upregulated proteins, and the core proteins were mostly related to translation, ribosomal structure and biogenesis.

### 3.5. Transcriptional analysis of DEPs

To test the correspondence between transcript levels and protein expression levels, the transcript levels of 9 DEPs were measured by qPCR, and the result are shown in Table 1. The nine genes represent the predominant metabolic pathways of energy production and conversion, carbohydrate transport and metabolism, post-translational modification, cell wall biogenesis and inorganic ion transport of *B. pumilus* and *K. vulgare*, respectively, which showed similar trends in terms of changes in their transcript levels and protein expression levels.

**Table 1:** Transcriptional complementation of the proteomic output by qPCR assays.

Gene symbol	Gene product	Fold change (qPCR)^*^	Fold change (proteomics)^*^
*tsf*	Elongation factor Ts	1.683	1.205
*ccpA*	Catabolite control protein A	0.338	0.795
*ftsY*	Signal recognition particle receptor FtsY	1.672	1.480
*clpC*	Negative regulator of genetic competence ClpC/MecB	0.874	0.779
*dpp*	Oligopeptide/dipeptide ABC transporter, ATPase subunit	0.547	0.737
*mglA3*	Suger ABC-transporter, ATPase component	0.733	0.707
*ubiE*	Ubiquinone/menaquinone biosynthesis C-methyltransferase	0.840	0.870
*nolF*/*acrA*	Efflux transporter, RND family, MFP subunit	0.717	0.856
*greA*	Transcription elongation factor	1.378	1.141

*Gene expression ratio of 18 h to 40 h of *B. pumilus* SY-A9 or *K. vulgare* 25B-1 in the co-culture fermentation system. The expression of 16S rRNA was used as an internal control.

## 4. Discussion

To explore the reasons for inhibiting the growth of *B. pumilus*, iTRAQ-based proteomic analysis was used in a coculture fermentation system, and a total of 178 DEPs were obtained in the B18 vs. B40 and K18 vs. K40.

Twelve DEPs of *B. pumilus*, including M5P1Y9, G4EPA3, A0A0C5CAF1, Q3EJU2, M4KMP7, W7R788, L8ASG9, A0A0C5CB21, A0A0K6MRG1, A0A0C5CMU8, Q9KEE6, and A0A132BIK5, were predicted to be involved in the response to acid stress. Here, the acid resistance mechanisms of *B. pumilus* including the general stress response, pH homeostasis maintenance, metabolic rearrangements and alkali production, and the secondary oxidative stress response are discussed. In Gram-positive bacteria, the alternative sigma factor σ^B^, as the key sigma factor, plays a vital role in regulating the expression of approximately 150 genes in response to significant changes in the surroundings and appear to control, at least in part, the general stress response (Desriac et al., 2013; Rothstein et al., 2017). In general, σ^B^ is activated by several stress conditions, e.g., heat shock, acid shock, NaCl or H_2_O_2_ exposure and ethanol shock and the activity is controlled by the regulator of Sigma B (RsbRST) complex (Yang et al., 1996; Chaturongakul and Boor, 2004; Lee et al., 2004; Ramesh et al., 2021). In the RsbRST-complex, serine/threonine-protein kinase (RsbT, M5P1Y9) acts as a switch that primarily receives stress signals to regulate the activity of σ^B^ (Ramesh et al., 2021). In this study, RsbT was downregulated in B18 vs. B40, indicating that *B. pumilus* could be under environmental stresses such as osmotic stress, oxidative stress, and acid stress in the coculture fermentation system at 40 h. Moreover, the expression of proteins regulated by σ^B^ was downregulated, such as general stress protein 30 (YxaB, G4EPA3), general stress protein 39 (YdaD, A0A0C5CAF1), glucose 1-dehydrogenase (Gdh, Q3EJU2), and uncharacterized oxidoreductase (YccK, M4KMP4). The negative regulator of genetic competence ClpC/MecB (ClpC, L8ASG9) is reported to be required for various processes, including the stress response, sporulation and competence in *B. subtilis* (Singh et al., 2015), whereas ATP-dependent Clp protease ATP-binding subunit (ClpE, W7R788) acts synergistically with ClpC (Nair et al., 1999). Therefore, the downregulation of ClpC and ClpE also indicated that *B. pumilus* was under oxidative stress and acid stress in the coculture fermentation system.

Under acidic conditions, bacteria activate enzymes such as F_o_F_1_-ATPase, proton transporter, and amino-acid decarboxylase systems to maintain intracellular pH homeostasis (Mols and Abee, 2011). In aerobic bacteria, active H^+^ transport is integrated with electron transport in the respiratory chain, while anaerobic bacteria use ATP hydrolysis for H^+^-ATPase molecules (Gandhi and Chikindas, 2007). *B. pumilus*, as a facultative anaerobic bacterium, may use both ATP synthesis and hydrolysis to maintain its pH homeostasis. The NADH dehydrogenase-like protein (YumB, M4KYK9) involved in electron transport in the respiratory chain was downregulated, and the nucleotide-binding protein (YvcJ, A0A0C5C8R2) that displays ATPase activity was also downregulated. The downregulation of these two DEPs suggested that *B. pumilus* could use both ATP synthesis and hydrolysis to regulate and thereby maintain intracellular pH homeostasis.

Under acidic conditions, alkali production is observed, such as urea, arginine and ammonia (Desriac et al., 2013). Urea and arginine are rapidly hydrolyzed to ammonia, which then combines with H^+^ to produce NH_4_^+^ to raise the internal pH (Lemos and Burne, 2008). Moreover, it has been reported that up regulation of the acid-inducible glycine decarboxylase system (GcvPA, GcvPB, and GcvT) can produce ammonia and assist in counteracting cytoplasmic acidification (Okamura-Ikeda et al., 1993). The expression of the enzyme catalyzing pyruvate to acetoin was induced upon exposure to acid shocks (Kitko et al., 2009; Mols and Abee, 2011). Here, acetoin: 2,6-dichlorophenolindophenol oxidoreductase subunit alpha (AcoA, A0A0K6MRG1), which cleaves acetoin into acetate and acetaldehyde, was upregulated. The probable glycine dehydrogenase (GcvPB, A0A0C5CMU8) was also upregulated. The upregulation of AcoA and GcvPB suggested that *B. pumilus* was under acid shocks at 18 h in the coculture fermentation system and could produce ammonia in response to acid stress.

Gram-positive bacteria exposed to inorganic and organic acids under aerobic conditions have been reported to show a major oxidative response (Desriac et al., 2013), and the products of oxygen include superoxide, hydroxyl radicals, and hydrogen peroxide. To survive the toxic effects of reactive oxygen species (ROS), the secondary oxidative stress response upon exposure to acid shocks is indicated by the induction of oxidative stress-associated genes, such as thioredoxins, catalases, and superoxide dismutase (Boylan et al., 2008; Mols et al., 2010). In this paper, upregulation of catalase-peroxidase (KatG, Q9KEE6) indicated that *B. pumilus* induced KatG to scavenge intracellular H_2_O_2_ in response to oxidative stress and acid shocks. In addition, the redox-sensing transcriptional repressor (Rex, A0A0132BIK5), which controls the respiratory pathway by regulating transcription in response to changes in the cellular NADH/NAD^+^ radox state, was downregulated (Park et al., 2018). The downregulation of Rex indicated that *B. pumilus* was under acid stress and could resist acid shock.

2-KLG, the acidic and oxidation product of *K. vulgare*, has been reported to cause oxidative stress, osmotic stress, and DNA damage in *Gluconobacter oxydans* (Fang et al., 2021). In this paper, it was found that *B. pumilus* might be under acid shocks from *K. vulgare.* In addition, based on proteomics analysis, 8 DEPs of *K. vulgare*, including E3F3W9, E3EYA2, A0A1B1VM42, F9Y8N8, A0A1B1VND4, E3EZ83, E3EZI0, and A0A1B1VNF7, were associated with quorum sensing (QS) and virulence. The LysR-type transcriptional regulator (KVU_1790, E3F3W9) was upregulated and regulated a diverse set of genes involved in virulence, metabolism, QS and motility (Maddocks and Oyston, 2008). The GntR-type transcriptional regulators (KVU_2493, E3EYA2; KVU_0514, A0A1B1VM42) were downregulated and are capable of regulating a variety of biological processes, including metabolic pathways, morphogenesis, the cell envelope stress response and antibiotic production (Lemmens et al., 2019). The ATP-dependent Clp protease proteolytic subunit (ClpP, A0A1B1VND4) was upregulated, which degrades or hydrolyzes misfolded or damaged proteins in response to environmental stress and regulates the production of virulence factors (Michel et al., 2006; Cohn et al., 2007). The upregulation of ClpP indicated that *K. vulgare* might be under environmental stress in the coculture fermentation system and produce virulence factors in response to it. Interestingly, involved in multidrug efflux proteins, ABC-type multidrug transport system ATPase component-like protein (KVU_2149, E3EZI0), efflux transporter, RND family, MFP subunit (AcrA, A0A1B1VNF7), and oligopeptide/dipeptide (Opp/Dpp) ABC transporter, ATPase subunit (KVU_1262, E3EZ83) were downregulated. In Gram-negative bacteria, the multidrug efflux mechanism is mediated by transport proteins that are resistant to toxic compounds, and these transporters are associated with intrinsic and acquired antimicrobial resistance (Moreira et al., 2004). In addition, KVU_1262 is reported to be involved in the transport of amino acids, virulence factors, and QS factors (Masukagami et al., 2018). The CRISPR-associated protein (KVU_1368, F9Y8N8) correlated with QS (Hoyland-Kroghsbo et al., 2017) was upregulated, implying the presence of a QS system in the coculture fermentation system at 18 h.

The QS system is known as a cell–cell communication mechanism that is mediated by cell density and involved in the production, release, and detection of extracellular signal molecules (autoinducers, AI) (Hoyland-Kroghsbo et al., 2017). When the threshold concentration of AI is reached and detected by the group, a population-wide alteration in gene expression will occur in response to it. QS is a mechanism that controls virulence factor secretion, competence, biofilm formation, bioluminescence, and sporulation (Bassler and Losick, 2006). In general, N-acylhomoserine lactones (AHLs) are one of the AIs used by Gram-negative bacteria (Kaufmann et al., 2005). However, the probable quorum-quenching lactonase (YtnP, D4G066) of *B. pumilus* was downregulated, which was reported to hydrolyze AI to inhibit the signaling pathway, biofilm formation, and production of virulence factors (Sun et al., 2021). In vitamin C fermentation, Wang et al. (2019) established the programmed cell death module based on the AHLs-LuxR QS system in *G. oxydans* to reduce the competition between *G. oxydans* and *K. vulgare*, but not mentioned the QS system of *K. vulgare*. Although the QS system in other Gram-negative bacteria is well defined, such as *Pseudomonas aeruginosa*, *Vibrio fischeri*, and *Vibrio harveyi*, it is not clear what kind of autoinducers are produced by *K. vulgare*. Moreover, it is not known whether *K. vulgare* produces virulence factors, and the relationship between virulence factors and autoinducers in the QS system of *K. vulgare* has rarely been mentioned.

In our previous study, we found that *K. vulgare* contains an N-acyl-L-homoserine lactone synthetase-like protein (KVU_2075) and LuxR (KVU_2076), indicating that *K. vulgare* could use the AHLs-LuxR QS system to regulate its own cell density (Zhang and Lyu, 2022). Here, to further investigate the availability of QS in the coculture fermentation system, the expression of genes *KVU_2075* (encoded N-acyl-L-homoserine lactone synthetase-like protein), *KVU_2076* (encoded LuxR family transcriptional regulator), and *ytnP* (encoded probable quorum-quenching lactonase) were measured in monoculture and coculture fermentation systems at 6, 18, 40, and 72 h, respectively (Supplementary Figure S1). The expression of genes *KVU_2075* and *KVU_2076* of *K. vulgare* in the coculture fermentation system was higher than that in the monoculture fermentation system at 6 h and 18 h. The high expression of *KVU_2075* implies a high cell density of *K. vulgare* in the coculture fermentation system (Subramoni and Vittorio, 2009). However, at 40 h and 72 h, the expression of *K. vulgare* genes *KVU_2075* and *KVU_2076* in the coculture fermentation system was significantly lower than that in the monoculture fermentation system. At 6 h and 40 h, no expression levels of the *ytnP* gene of *B. pumilus* were detected in either monoculture or coculture fermentation systems, whereas at 18 h and 72 h, *ytnP* expression levels were much higher in the coculture fermentation system than in the monoculture fermentation system. The lower expression of *KVU_2075* and *KVU_2076* and the higher expression of *ytnP* suggested that *B. pumilus* could secrete YtnP to hydrolyze the AHLs produced by *K. vulgare* to interfere with the normal signaling pathway of *K. vulgare* in the coculture fermentation system.

**Figure 5:**
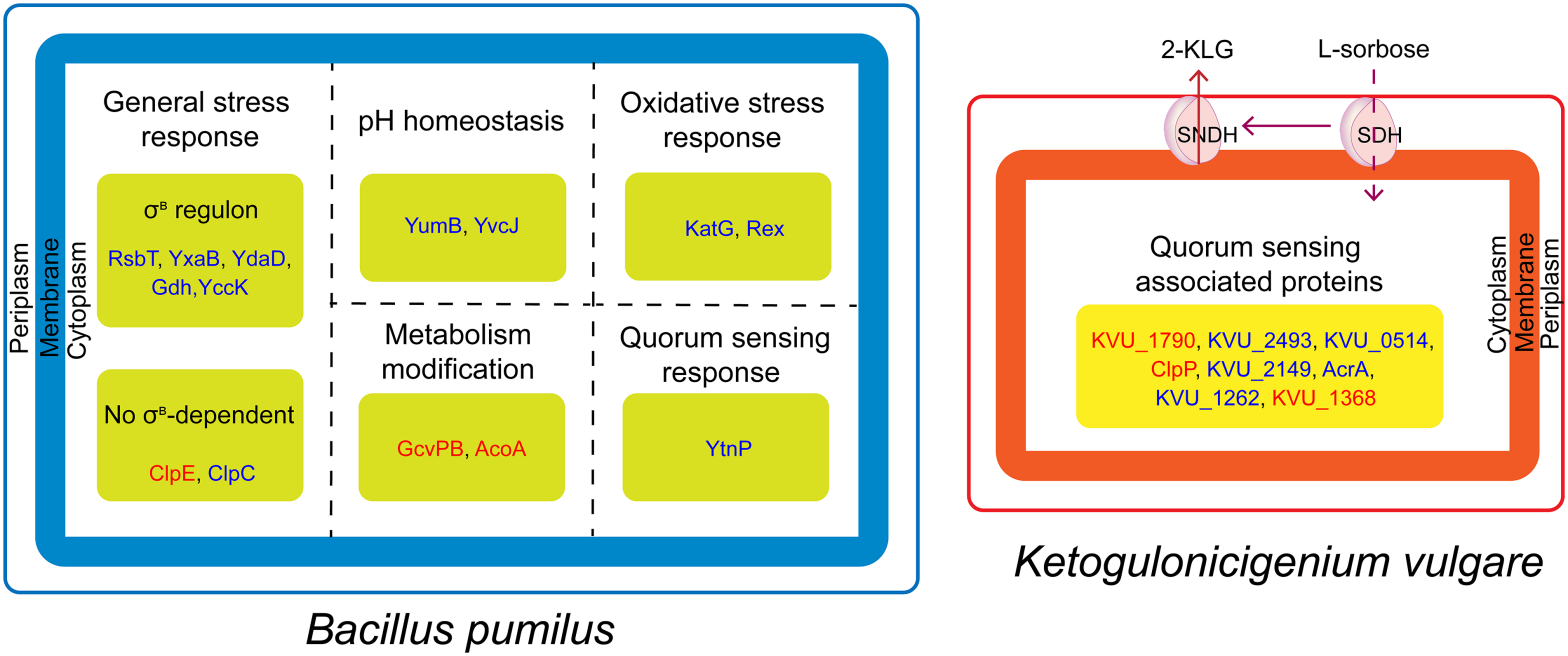
Schematic representation of antagonistic mechanism between *B. pumilus* and *K. vulgare*. *K. vulgare* produce 2-KLG and quorum sensing associated virulence factors against *B. pumilus*, whereas, *B. pumilus* produce proteins in response to acid stress and quorum sensing. The red and blue fonts represent upregulated and downregulated proteins, respectively.

In this paper, we analyzed the reasons for inhibiting the growth of *B. pumilus* by iTRAQ-based proteomics analysis. In conclusion, *B. pumilus* might be under the acid shock and quorum sensing system of *K. vulgare* and the relationship between the two bacteria could be antagonistic (Figure 5). In addition, the synthetic consortium used in this study was screened from soil. Thus, the study of the interactions between *K. vulgare* and *B. pumilus* not only provides a better insight into the interactions between synthetic consortia in vitamin C microbial fermentation, but also further contributes to the understanding of soil-microbe interactions.

## Data availability statement

The datasets presented in this study can be found in online repositories. The names of the repository/repositories and accession number(s) can be found in the article/Supplementary material.

## Author contributions

SL contributed to conception and design of the study. QZ performed the statistical analysis and wrote the first draft of the manuscript. All authors contributed to manuscript revision, read, and approved the submitted version.

## Funding

This work was supported by the National Science Foundation of China (Grant number: 31370077) and Liaoning Province Science Research Fund (Grant number: LSNJC201915). Shenyang Agricultural University Postgraduate Innovation Cultivation Project (Grant number: 2022YCXB11).

## Conflict of interest

The authors declare that the research was conducted in the absence of any commercial or financial relationships that could be construed as a potential conflict of interest.

## Publisher’s note

All claims expressed in this article are solely those of the authors and do not necessarily represent those of their affiliated organizations, or those of the publisher, the editors and the reviewers. Any product that may be evaluated in this article, or claim that may be made by its manufacturer, is not guaranteed or endorsed by the publisher.

## Footnotes

^1^https://blast.ncbi.nlm.nih.gov/Blast.cgi/

^2^https://www.genome.jp/kegg/

^3^https://www.string-db.org
