# Supplementary material for: iTRAQ-based proteomics analysis of Bacillus pumilus responses to acid stress and quorum sensing in a vitamin C fermentation system
Source: Front Microbiol. 2023 Mar 21;14:1131000. doi: 10.3389/fmicb.2023.1131000 (PMC10070982; doi:10.3389/fmicb.2023.1131000)
Supplement: Supplementary file 1 [file Data_Sheet_1.docx]

Supplementary Material

iTRAQ-based proteomics analysis of *Bacillus pumilus* responses to acid stress and quorum sensing in a vitamin C fermentation system

**Qian Zhang^1^, Shuxia Lyu^1*^**

*** Correspondence:** Shuxia Lyu: lushuxia@syau.edu.cn

# Supplementary Tables

## Supplementary Table 1

Table S1 qPCR primers used in the study

| Strain | Gene product | Gene symbol | Primer name | Primers sequence（5'→3'） |
| --- | --- | --- | --- | --- |
| *B. p* | Elongation factor Ts | *tsf* | *tsf*_F | AAATTACGCTGCGCCGTTTC |
|  |  |  | *tsf*_R | GGGTTTACAGCAGCAACGTG |
| *B. p* | Catabolite control protein A | *ccpA* | *ccpA* _F | CCTAGCGCCATTTCGTCAGT |
|  |  |  | *ccpA* _R | CGCTTGTTGCTGAAGGAGAC |
| *B. p* | Signal recognition particle receptor FtsY | *ftsY* | *ftsY _F* | TTTGTAGGCGTGAACGGTGT |
|  |  |  | *ftsY _R* | AACGACAGGAACACCAGAGC |
| *B. p* | Negative regulator of genetic competence ClpC/MecB | *clpC* | *clpC* _F | CGTGCGTTTAGACCGGAGT |
|  |  |  | *clpC* _R | GTCCTGCTCTTTTAGTCGTTTTGT |
| *B. p* | Probable quorum-quenching lactonase | *ytnP* | *ytnP_F* | ATGCCAAGACCCAAAGCGG |
|  |  |  | *ytnP_R* | CGCTCACAAGTCAGGGAGAAA |
| *B. p* | 16S Ribosomal RNA | *16S rRNA* | *16S rRNA* _F | GTGGGGTAATGGCTCACCAA |
|  |  |  | *16S rRNA* _R | GATTCCCTACTGCTGCCTCC |
| *K. v* | Oligopeptide/dipeptide ABC transporter, ATPase subunit | *dpp* | *dpp* _F | ACGGGCAGGTGATCTTTGAC |
|  |  |  | *dpp*_R | GTGGCTACGGATGTTCTCGG |
| *K. v* | Suger ABC-transporter, ATPase component | *mglA3* | *mglA3*_F | TGTCGGAACTGATGGCTCTG |
|  |  |  | *mglA3*_R | GCGTGAGACCCTCGGTATTT |
| *K. v* | Ubiquinone/menaquinone biosynthesis C-methyltransferase | *ubiE* | *ubiE _F* | CGCTCGGTCGCCAGTAAATA |
|  |  |  | *ubiE _R* | GCCAGTCCATCATCGCATCT |
| *K. v* | Efflux transporter, RND family, MFP subunit | *nolF*/*acrA* | *nolF* _F | ATCGTCACCGCAAACCAAAC |
|  |  |  | *nolF* _R | CATCATCCAGCGTCAGGGTC |
| *K. v* | Transcription elongation factor | *greA* | *greA* _F | GCACCTGAAATCTGTCGAGC |
|  |  |  | *greA* _R | TAACGCCCTCCAGCTCTTTG |
| *K. v* | N-acyl-L-homoserine lactone synthetase-like protein | *KVU_2075* | *KVU_2075_*F | ATTTTGCTGATCCCCTCGCT |
|  |  |  | *KVU_2075_*R | GCTTTGTCTCGAACGATCTGC |
| *K. v* | LuxR family transcriptional regulator | *KVU_2076* | *KVU_2076_*F | TTCTACGCACTCCACATCCG |
|  |  |  | *KVU_2076_*R | GGAAGGTCGATTTCGCTCCA |
| *K. v* | 16S Ribosomal RNA | *16S rRNA* | *16S rRNA* _F | TTGCCGGACTTAACCGAACA |
|  |  |  | *16S rRNA* _R | CGCAGAACCTTACCAACCCT |

## Supplementary Table 2

Table S2 COG functional annotation of the up- and downregulated DEPs of *B. pumilus* SY-A9

| COG functional classification | Accession | Description | Treatment combination | Fold change |
| --- | --- | --- | --- | --- |
| **Information storage and processing** | | | | |
| **[J] Translation, ribosomal structure and biogenesis** | A0A0K6MFS2 | 50S ribosomal protein L24 | B18 vs. B40 | 1.211 |
|  | A0A0K6MGM2 | 50S ribosomal protein L15 | B18 vs. B40 | 1.283 |
|  | A0A0A0TRL9 | Polyribonucleotide nucleotidyltransferase | B18 vs. B40 | 1.277 |
|  | A0A0K6MKL6 | Valine--tRNA ligase | B18 vs. B40 | 0.811 |
|  | Q9KA77 | Translation initiation factor IF-2 | B18 vs. B40 | 0.804 |
|  | A0A132BER5 | Threonine--tRNA ligase 1 | B18 vs. B40 | 0.784 |
|  | A0A164SUI4 | Tryptophan--tRNA ligase | B18 vs. B40 | 0.701 |
| **[K] Transcription** | A0A0D1IA59 | Transcriptional regulatory protein DegU | B18 vs. B40 | 0.821 |
|  | A0A150KVB1 | Transcription termination factor Rho | B18 vs. B40 | 0.810 |
|  | A0A132BIK5 | Redox-sensing transcriptional repressor Rex | B18 vs. B40 | 0.793 |
|  | R9U0I2 | Probable licABCH operon regulator | B18 vs. B40 | 0.715 |
|  | D4G3E3 | Stage V sporulation protein T | B18 vs. B40 | 0.541 |
| **[L] Replication, recombination and repair** | Q3ER61 | DNA polymerase I | B18 vs. B40 | 0.693 |
| **Cellular processes and signaling** | | | | |
| **[D] Cell cycle control, cell division, chromosome partitioning** | A0A0K6MCF3 | Cell division protein FtsZ | B18 vs. B40 | 0.793 |
| **[T] Signal transduction mechanisms** | A0A063XD71 | Anti-sigma F factor | B18 vs. B40 | 1.214 |
|  | A0A150KVB5 | Sporulation initiation phosphotransferase F | B18 vs. B40 | 1.268 |
|  | M5P1Y9 | Serine/threonine-protein kinase RsbT | B18 vs. B40 | 0.735 |
| **[M] Cell wall/ membrane/ envelope biogenesis** | D4G066 | Probable quorum-quenching lactonase YtnP | B18 vs. B40 | 0.698 |
|  | G4EPA3 | General stress protein 30 | B18 vs. B40 | 0.718 |
| **[U] Intracellular trafficking, secretion, and vesicular transport** | A0A0C5CCU5 | Signal recognition particle receptor FtsY | B18 vs. B40 | 1.480 |
|  | Q9KA10 | Signal recognition particle protein | B18 vs. B40 | 0.801 |
| **[O] Posttranslational modification, protein turnover, chaperones** | W7R788 | ATP-dependent Clp protease ATP-binding subunit ClpE | B18 vs. B40 | 1.359 |
|  | L8ASG9 | Negative regulator of genetic competence ClpC/MecB | B18 vs. B40 | 0.779 |
| **Metabolism** | | | | |
| **[C] Energy production and conversion** | M5PE37 | Succinate--CoA ligase [ADP-forming] subunit alpha | B18 vs. B40 | 1.473 |
|  | Q9KA21 |  | B18 vs. B40 | 1.609 |
|  | A0A0A0TUR6 | Sulfite reductase [NADPH] hemoprotein beta-component | B18 vs. B40 | 1.253 |
|  | A0A080UR44 | 1-pyrroline-5-carboxylate dehydrogenase | B18 vs. B40 | 1.578 |
|  | A0A0C5C9Y6 |  | B18 vs. B40 | 1.752 |
|  | A0A0K6JRL3 | Alpha-ketoglutaric semialdehyde dehydrogenase | B18 vs. B40 | 1.221 |
|  | Q9KAH5 | Malonate-semialdehyde dehydrogenase | B18 vs. B40 | 1.225 |
|  | A0A1B2AYJ1 | Malate dehydrogenase | B18 vs. B40 | 1.208 |
|  | A0A0C5C4W8 | Succinate--CoA ligase [ADP-forming] subunit beta | B18 vs. B40 | 1.732 |
|  | A0A0K6MGU1 |  | B18 vs. B40 | 1.513 |
|  | M5P4D5 |  | B18 vs. B40 | 1.677 |
|  | A0A0C5C637 | Isocitrate dehydrogenase [NADP] | B18 vs. B40 | 1.301 |
|  | M5P776 |  | B18 vs. B40 | 1.262 |
|  | A0A0C5CC70 | Aconitate/2-methylaconitate hydratase | B18 vs. B40 | 1.289 |
|  | A0A0W8JR07 |  | B18 vs. B40 | 1.310 |
|  | Q9KF76 | Sulfite reductase [NADPH] flavoprotein alpha-component | B18 vs. B40 | 1.335 |
|  | A0A0K6MRG1 | Acetoin:2,6-dichlorophenolindophenol oxidoreductase subunit alpha | B18 vs. B40 | 1.640 |
|  | I2C162 | 1-pyrroline-5-carboxylate dehydrogenase 2 | B18 vs. B40 | 1.919 |
|  | M4KYK9 | NADH dehydrogenase-like protein YumB | B18 vs. B40 | 0.740 |
|  | A0A0W8KB03 | Dihydrolipoyllysine-residue succinyltransferase component of 2-oxoglutarate dehydrogenase complex | B18 vs. B40 | 0.686 |
|  | A0A0A0U0S2 | Aldo-keto reductase IolS | B18 vs. B40 | 0.757 |
|  | M4KMP7 | Uncharacterized oxidoreductase YccK | B18 vs. B40 | 0.744 |
|  | A0A0K6MNE9 | Dihydrolipoyl dehydrogenase | B18 vs. B40 | 0.783 |
|  | M5P4V0 |  | B18 vs. B40 | 0.742 |
|  | A0A0H3DYP3 | Pyruvate dehydrogenase E1 component subunit alpha | B18 vs. B40 | 0.792 |
|  | E0U3X7 | Pyruvate dehydrogenase E1 component subunit beta | B18 vs. B40 | 0.715 |
|  | A0A080UGF8 | Dihydrolipoyllysine-residue acetyltransferase component of pyruvate dehydrogenase complex | B18 vs. B40 | 0.524 |
| **[G] Carbohydrate transport and metabolism** | A0A080UNH5 | Probable 6-phospho-beta-glucosidase | B18 vs. B40 | 1.408 |
|  | A0A0W8K7W6 | Altronate dehydratase | B18 vs. B40 | 0.814 |
|  | D4G066 | Probable quorum-quenching lactonase YtnP | B18 vs. B40 | 0.698 |
|  | G4EPA3 | General stress protein 30 | B18 vs. B40 | 0.718 |
| **[E] Amino acid transport and metabolism** | I2C439 | 5-methyltetrahydropteroyltriglutamate--homocysteine methyltransferase | B18 vs. B40 | 2.136 |
|  | A0A0D7XU75 | Histidinol dehydrogenase | B18 vs. B40 | 1.230 |
|  | L8APV3 | Tryptophan synthase beta chain | B18 vs. B40 | 1.343 |
|  | A0A0C5CMU8 | Probable glycine dehydrogenase (decarboxylating) subunit 2 | B18 vs. B40 | 1.240 |
|  | Q9K8F0 | 3-isopropylmalate dehydratase large subunit | B18 vs. B40 | 1.638 |
|  | Q3EX80 | Ketol-acid reductoisomerase (NADP(+)) | B18 vs. B40 | 1.473 |
|  | I2C928 | Protein AroA(G) | B18 vs. B40 | 1.735 |
|  | A0A0D1JHT5 | Oligopeptide-binding protein OppA | B18 vs. B40 | 0.804 |
|  | A0A0K6K705 | Ornithine aminotransferase | B18 vs. B40 | 0.820 |
|  | A0A0K6MHU6 |  | B18 vs. B40 | 0.772 |
| **[F] Nucleotide transport and metabolism** | A0A0K6MEM5 | Bifunctional purine biosynthesis protein PurH | B18 vs. B40 | 0.780 |
| **[H] Coenzyme transport and metabolism** | Q3EX80 | Ketol-acid reductoisomerase (NADP(+)) | B18 vs. B40 | 1.473 |
|  | Q9KCL4 | 6,7-dimethyl-8-ribityllumazine synthase | B18 vs. B40 | 0.725 |
|  | M5PEA2 | Ribonuclease J1 | B18 vs. B40 | 0.789 |
|  | M5PE06 | Glutamate-1-semialdehyde 2,1-aminomutase | B18 vs. B40 | 0.801 |
| **[I] Lipid transport and metabolism** | A0A0A0TVE0 | Probable succinyl-CoA:3-ketoacid coenzyme A transferase subunit B | B18 vs. B40 | 1.278 |
|  | E1UR23 | 3-oxoacyl-[acyl-carrier-protein] synthase 2 | B18 vs. B40 | 1.345 |
|  | Q3EJU2 | Glucose 1-dehydrogenase | B18 vs. B40 | 0.810 |
|  | A0A0C5CAF1 | General stress protein 39 | B18 vs. B40 | 0.706 |
| **[P] Inorganic ion transport and metabolism** | Q9KCT2 | Sulfate adenylyltransferase | B18 vs. B40 | 1.851 |
|  | Q9KEE6 | Catalase-peroxidase | B18 vs. B40 | 0.784 |
| **[Q] Secondary metabolites biosynthesis, transport and catabolism** | Q3EJU2 | Glucose 1-dehydrogenase | B18 vs. B40 | 0.810 |
|  | A0A0C5CAF1 | General stress protein 39 | B18 vs. B40 | 0.706 |
| **Poorly characterized** | | | | |
| **[S] Function unknown** | A0A0K6N168 | FMN-dependent NADPH-azoreductase | B18 vs. B40 | 1.325 |
|  | A0A0C3KKU4 | UPF0234 protein yitk | B18 vs. B40 | 0.589 |
|  | A0A0C5CB21 | Nucleotide-binding protein YvcJ | B18 vs. B40 | 0.727 |
|  | A0A0H3E933 | Glyoxal reductase | B18 vs. B40 | 0.774 |
|  | A0A0C5C8R2 | Stage V sporulation protein S | B18 vs. B40 | 0.765 |
|  | A0A0K6MEX2 | Uncharacterized protein YneT | B18 vs. B40 | 0.717 |
|  | A0A0D1IR43 | Hydrolase YhcX | B18 vs. B40 | 0.774 |
| **No annotation** | A0A0C5C8W7 | Elongation factor Ts | B18 vs. B40 | 1.205 |
|  | I2C927 | Catabolite control protein A | B18 vs. B40 | 0.796 |
|  | G4EW54 | Uncharacterized protein | B18 vs. B40 | 0.808 |

## Supplementary Table 3

Table S3 COG functional annotation of the up- and downregulated DEPs of *K. vulgare* 25B-1

| COG functional classification | Accession | Description | Treatment combination | Fold change |
| --- | --- | --- | --- | --- |
| **Information storage and processing** | | | | |
| **[J] Translation, ribosomal structure and biogenesis** | F9Y6Y4 | 30S ribosomal protein S9 (*rpsI*) | K18 vs. K40 | 1.107 |
|  | F9Y5D7 | 50S ribosomal protein L3 (*rplC*) | K18 vs. K40 | 1.176 |
|  | F9Y5D2 | 50S ribosomal protein L22 (*rplV*) | K18 vs. K40 | 1.212 |
|  | F9Y5D0 | 50S ribosomal protein L16 (*rplP*) | K18 vs. K40 | 1.157 |
|  | F9Y5C1 | 50S ribosomal protein L18 (*rplR*) | K18 vs. K40 | 1.170 |
|  | F9Y5C5 | 50S ribosomal protein L5 (*rplE*) | K18 vs. K40 | 1.144 |
|  | F9Y442 | 50S ribosomal protein L9 (*rplI*) | K18 vs. K40 | 1.240 |
|  | E3EZC2 | 50S ribosomal protein L24 (*rplX*) | K18 vs. K40 | 1.121 |
|  | E3EYZ1 | 30S ribosomal protein S6 (*rpsF*) | K18 vs. K40 | 1.156 |
|  | E3EYQ5 | 50S ribosomal protein L30 (*rpmD*) | K18 vs. K40 | 1.291 |
|  | A0A1B1VS01 | 50S ribosomal protein L28 (*rpmB*) | K18 vs. K40 | 1.114 |
|  | E3EYQ4 | 50S ribosomal protein L15 (*rplO*) | K18 vs. K40 | 1.201 |
|  | E3EXL8 | 50S ribosomal protein L20 (*rplT*) | K18 vs. K40 | 1.193 |
|  | A0A1B1VR09 | 30S ribosomal protein S15 (*rpsO*) | K18 vs. K40 | 1.169 |
|  | A0A1B1VQQ5 | 30S ribosomal protein S13 (*rpsM)* | K18 vs. K40 | 1.192 |
|  | F9Y500 | rRNA small subunit methyltransferase B (*KVU_0793*) | K18 vs. K40 | 0.876 |
|  | F9Y6U8 | Elongation factor P (*efp*) | K18 vs. K40 | 0.877 |
|  | E3EZQ8 | Ribosome-binding ATPase YchF (*ychF*) | K18 vs. K40 | 0.906 |
| **[K] Transcription** | F9Y8F7 | Transcription elongation factor GreA (*greA*) | K18 vs. K40 | 1.141 |
|  | E3F3W9 | Transcriptional regulator, LysR family protein (*KVU_1790*) | K18 vs. K40 | 1.114 |
|  | F9Y4K4 | CsbD-like protein (*KVU_0722*) | K18 vs. K40 | 0.746 |
|  | E3EYA2 | Transcriptional regulator protein, GntR family protein (*KVU_2493*) | K18 vs. K40 | 0.866 |
|  | A0A1B1VM42 | Transcriptional regulator, GntR family protein (*KVU_0514*) | K18 vs. K40 | 0.820 |
| **[L] Replication, recombination and repair** | F9Y4S8 | Hydrolase, TatD family protein (*KVU_1973*) | K18 vs. K40 | 0.899 |
|  | E3EYR6 | Exodeoxyribonuclease 7 small subunit (*xseB*) | K18 vs. K40 | 0.901 |
| **Cellular processes and signaling** | | | | |
| **[D] Cell cycle control, cell division, chromosome partitioning** | A0A1B1VQW4 | ParA family ATPase for plasmid partitioning (*parA*) | K18 vs. K40 | 0.852 |
| **[V] Defense mechanisms** | E3EZI0 | ABC-type multidrug transport system ATPase component-like protein (*KVU_2149*) | K18 vs. K40 | 0.857 |
| **[M] Cell wall/ membrane/ envelope biogenesis** | A0A1B1VNF7 | Efflux transporter, RND family, MFP subunit (*nolF*) | K18 vs. K40 | 0.856 |
|  | F9Y4C5 | Salt-stress induced outer membrane protein (*KVU_1899*) | K18 vs. K40 | 0.723 |
|  | A0A1B1VK02 | Putative lipoprotein (*KVU_2303*) | K18 vs. K40 | 0.790 |
|  | E3EX45 | OmpA/MotB domain protein (KVU_2440) | K18 vs. K40 | 0.790 |
| **[U] Intracellular trafficking, secretion, and vesicular transport** | F9Y4L1 | Phage terminase GpA (*KVU_0729*) | K18 vs. K40 | 1.822 |
|  | A0A1B1VND4 | ATP-dependent Clp protease proteolytic subunit (*clpP*) | K18 vs. K40 | 0.781 |
| **[O] Posttranslational modification, protein turnover, chaperones** | A0A1B1VL36 | Anhydro-N-acetylmuramic acid kinase (*anmK*) | K18 vs. K40 | 1.214 |
|  | F9Y4L1 | Phage terminase GpA (*KVU_0729*) | K18 vs. K40 | 1.822 |
|  | A0A1B1VND4 | ATP-dependent Clp protease proteolytic subunit (*clpP*) | K18 vs. K40 | 0.781 |
|  | E3EZ27 | Peptidyl-prolyl cis-trans isomerase (*ppiB*) | K18 vs. K40 | 0.870 |
|  | E3EWW1 | Band 7 protein (*KVU_2361*) | K18 vs. K40 | 0.830 |
| **Metabolism** | | | | |
| **[C] Energy production and conversion** | E3EX63 | Succinate--CoA ligase [ADP-forming] subunit beta (*sucC*) | K18 vs. K40 | 1.267 |
|  | E3F0I7 | Dehydrogenase cytochrome c subunit (*gadh2*) | K18 vs. K40 | 0.721 |
|  | E3EZ20 | Acetyltransferase component of pyruvate dehydrogenase complex (*pdhB*) | K18 vs. K40 | 0.767 |
|  | A0A1B1VNK7 | C4-dicarboxylate transport protein (*dctA*) | K18 vs. K40 | 0.745 |
|  | F9Y7J7 | Dihydrolipoyllysine-residue succinyltransferase component of 2-oxoglutarate dehydrogenase complex (*sucB*) | K18 vs. K40 | 0.867 |
|  | F9Y867 | Sorbitol dehydrogenase cytochrome c subunit (*sldC*) | K18 vs. K40 | 0.896 |
| **[G] Carbohydrate transport and metabolism** | F9YAI2 | Glyceraldehyde-3-phosphate dehydrogenase (*gapB*) | K18 vs. K40 | 1.101 |
|  | E3F3G7 | Ribose-5-phosphate isomerase A (*rpiA*) | K18 vs. K40 | 1.191 |
|  | E3EZ24 | Fructose-bisphosphate aldolase (*fbaB*) | K18 vs. K40 | 1.116 |
|  | F9Y7D8 | Phosphoglucomutase-1 (*pgm*) | K18 vs. K40 | 0.889 |
|  | E3F0C2 | TRAP family transporter, periplasmic substrate-binding subunit (*KVU_2212*) | K18 vs. K40 | 0.790 |
|  | A0A1B1VS29 | 2,3-bisphosphoglycerate-independent phosphoglycerate mutase (*gpmI*) | K18 vs. K40 | 0.895 |
| **[E] Amino acid transport and metabolism** | F9YA52 | Saccharopine dehydrogenase family protein (*KVU_1625*) | K18 vs. K40 | 1.140 |
|  | F9Y851 | Alcohol dehydrogenase GroES-like protein (*KVU_2498*) | K18 vs. K40 | 1.385 |
|  | F9Y481 | Acetolactate synthase (*ilvB*) | K18 vs. K40 | 1.255 |
|  | F9Y480 | Acetolactate synthase (*ilvH*) | K18 vs. K40 | 1.245 |
|  | E3F4K5 | 1-(5-phosphoribosyl)-5-[(5-phosphoribosylamino)methylideneamino] imidazole-4-carboxamide isomerase (*hisA*) | K18 vs. K40 | 1.124 |
|  | A0A1B1VPL2 | Glutamate synthase (Ferredoxin) (*gltB*) | K18 vs. K40 | 1.183 |
|  | A0A1B1VMQ4 | ABC transporter, periplasmic substrate-binding protein, putative (*KVU_0903*) | K18 vs. K40 | 1.111 |
|  | A0A1B1VJR8 | Branched-chain-amino-acid aminotransferase (*ilvE*) | K18 vs. K40 | 1.174 |
|  | F9Y6C4 | Phosphoribosyl-AMP cyclohydrolase (*hisI*) | K18 vs. K40 | 0.811 |
|  | F9Y655 | Methionine synthase, vitamin-B12 independent (*KVU_2213*) | K18 vs. K40 | 0.831 |
|  | E3EYD8 | Glyoxalase/bleomycin resistance protein/dioxygenase (*KVU_0591*) | K18 vs. K40 | 0.837 |
|  | A0A1B1VPJ3 | Glyoxalase/Bleomycin resistance protein/dioxygenase domain protein (*KVU_1712*) | K18 vs. K40 | 0.850 |
|  | A0A1B1VN40 | ABC transporter substrate-binding protein (Oligopeptide) (*KVU_1067*) | K18 vs. K40 | 0.774 |
|  | A0A1B1VLE5 | Glutamine synthetase family protein (*KVU_0291*) | K18 vs. K40 | 0.844 |
|  | A0A1B1VKP8 | N-formylglutamate amidohydrolase family protein (*KVU_2549*) | K18 vs. K40 | 0.819 |
| **[F] Nucleotide transport and metabolism** | F9Y6V0 | Nucleoside diphosphate kinase (*ndk*) | K18 vs. K40 | 1.112 |
|  | A0A1B1VQ45 | Inosine-uridine preferring nucleoside hydrolase (*KVU_1884*) | K18 vs. K40 | 1.628 |
|  | E3F0F0 | Ser/Thr protein phosphatase family protein (*KVU_0042*) | K18 vs. K40 | 0.809 |
|  | E3EYE5 | Phosphoribosylaminoimidazole-succinocarboxamide synthase (*purC*) | K18 vs. K40 | 0.874 |
| **[H] Coenzyme transport and metabolism** | F9Y481 | Acetolactate synthase (*ilvB*) | K18 vs. K40 | 1.255 |
| **[P] Inorganic ion transport and metabolism** | F9Y7Q5 | Carbonic anhydrase (*KVU_0032*) | K18 vs. K40 | 1.481 |
|  | F9Y8T5 | Twin-arginine translocation pathway signal (*KVU_2573*) | K18 vs. K40 | 1.112 |
|  | E3EZ83 | Oligopeptide/dipeptide ABC transporter, ATPase subunit (*KVU_1262*) | K18 vs. K40 | 0.737 |
|  | E3EX13 | Sulfurtransferase (*sseA1*) | K18 vs. K40 | 0.894 |
|  | A0A1B1VPS8 | Catalase-peroxidase (*katA*) | K18 vs. K40 | 0.781 |
|  | A0A1B1VLX6 | Sugar ABC-transporter, ATP-ase component (*mglA3*) | K18 vs. K40 | 0.707 |
| **[Q] Secondary metabolites biosynthesis, transport and catabolism** | F9Y4E5 | Ubiquinone/menaquinone biosynthesis C-methyltransferase UbiE (*ubiE*) | K18 vs. K40 | 0.870 |
| **Poorly characterized** | | | | |
| **[S] Function unknown** | E3F0T3 | Uncharacterized protein (*KVU_0858*) | K18 vs. K40 | 1.115 |
|  | E3EZG9 | Uncharacterized protein (*KVU_2139*) | K18 vs. K40 | 1.140 |
|  | A0A1B1VMU4 | Uncharacterized protein (*KVU_0908*) | K18 vs. K40 | 1.102 |
|  | F9Y8B8 | Nucleotide-binding protein KVU_2565 (*KVU_2565*) | K18 vs. K40 | 0.867 |
|  | F9Y6H3 | Short-chain dehydrogenase/reductase SDR (*KVU_1080*) | K18 vs. K40 | 0.781 |
|  | E3F493 | Uncharacterized protein (*KVU_0468*) | K18 vs. K40 | 0.757 |
|  | A0A1B1VLG9 | Outer membrane protein, 28 kDa (*KVU_0362*) | K18 vs. K40 | 0.867 |
|  | A0A1B1VLD7 | Uncharacterized protein (*KVU_0325*) | K18 vs. K40 | 0.753 |
| **No annotation** | F9Y9L4 | Uncharacterized protein (*KVU_1513*) | K18 vs. K40 | 1.170 |
|  | F9Y8N8 | CRISPR-associated protein, Csd5d family protein (*KVU_1368*) | K18 vs. K40 | 1.877 |
|  | E3EYT6 | Glycerol-3-phosphate dehydrogenase (*KVU_2522*) | K18 vs. K40 | 1.171 |
|  | A0A1B1VS48 | Uncharacterized protein (*KVU_1981*) | K18 vs. K40 | 1.122 |
|  | A0A1B1VL98 | Uncharacterized protein (*KVU_0282*) | K18 vs. K40 | 1.136 |
|  | A0A1B1VK72 | Trehalose-6-phosphate synthase (*otsA*) | K18 vs. K40 | 1.125 |
|  | E3F131 | Probable branched-chain-amino-acid aminotransferase (*ilvE*) | K18 vs. K40 | 1.156 |
|  | E3F484 | 10 kDa chaperonin (*groS*) | K18 vs. K40 | 1.160 |
|  | F9Y8F8 | Uncharacterized protein (*KVU_0129*) | K18 vs. K40 | 0.869 |
|  | F9Y865 | Uncharacterized protein (*KVU_2512*) | K18 vs. K40 | 0.843 |
|  | F9Y6C1 | Uncharacterized protein (*KVU_1028*) | K18 vs. K40 | 0.632 |
|  | E3F1U5 | Uncharacterized protein (*KVU_1603*) | K18 vs. K40 | 0.776 |
|  | A0A1B1VKY0 | Methionine aminopeptidase (*map*) | K18 vs. K40 | 0.894 |

# Supplementary Figures

**
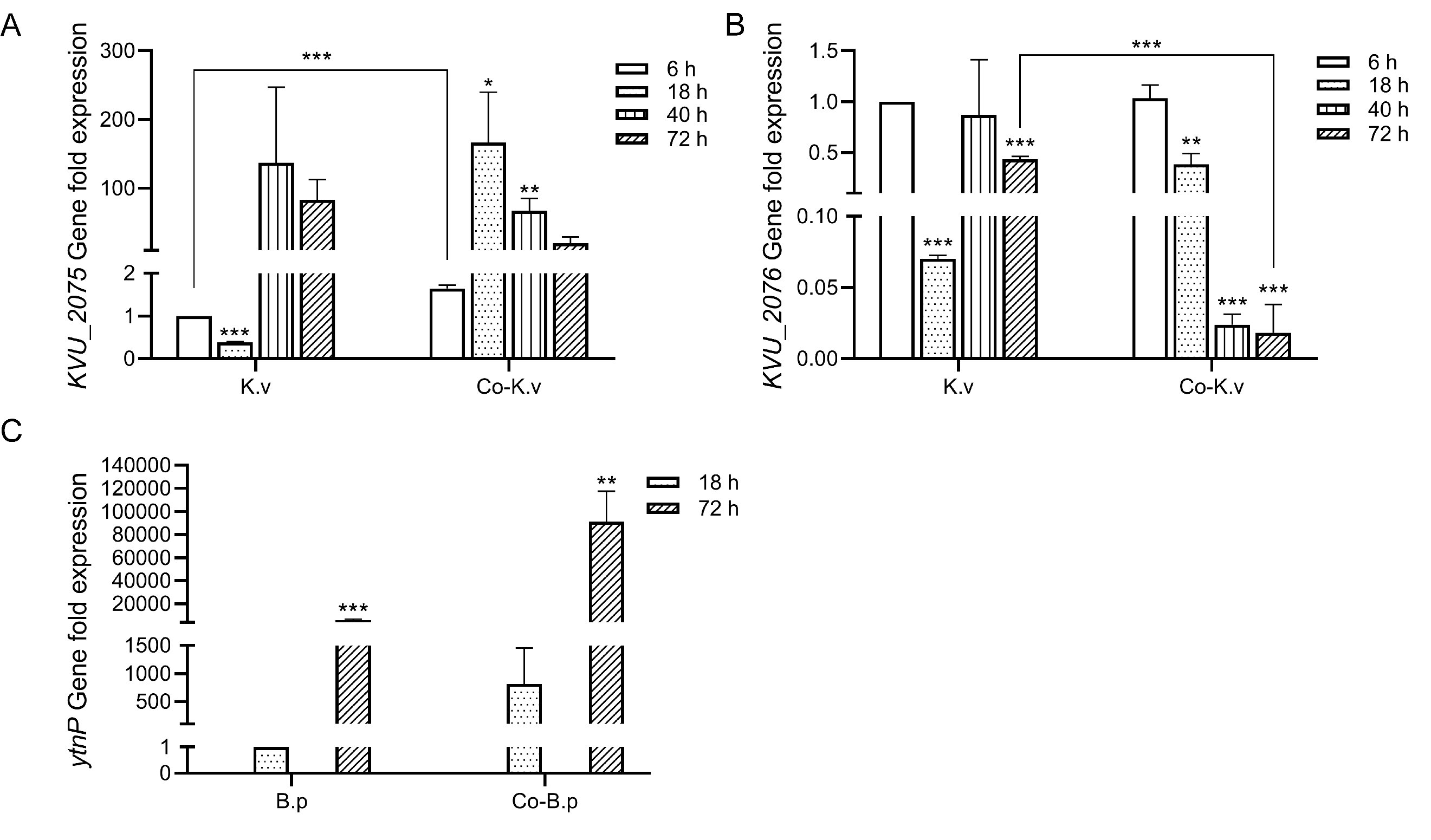
**

**Supplementary Figure 1.** Expression levels of quorum sensing-related genes in Vc fermentation systems at 6, 18, 40, and 72 h, respectively. K.v, *K. vulgare* in the monoculture fermentation system; Co-K.v, *K. vulgare* in the coculture fermentation system; B.p, *B. pumilus* in the monoculture fermentation system; Co-B.p, *B. pumilus* in the coculture fermentation system. *KVU_2075* encodes N-acyl-L-homoserine lactone synthetase-like protein **(A)**, *KVU_2076* encodes LuxR family transcriptional regulator **(B)**, and *ytnP* encodes probable quorum-quenching lactonase **(C)**
